# Detection, cerebrovascular complications and risk factors associated with vertebrobasilar dolichoectasia: a scoping review

**DOI:** 10.3389/fneur.2025.1668912

**Published:** 2025-10-15

**Authors:** Juan Morales-Verdugo, Francisco Pérez-Rojas, Alan Figueroa-Figueroa, Javier Lagos-Fica, Joaquín Vera-Paredes, Olivia García-Suárez, Juan Cabezas-Salgado, Félix Orellana-Cortés

**Affiliations:** ^1^Departamento de Ciencias Preclínicas, Facultad de Medicina, Universidad Católica del Maule, Talca, Chile; ^2^Departamento de Morfología y Biología Celular, Grupo SINPOS, Universidad de Oviedo, Oviedo, Spain; ^3^Escuela de Medicina, Facultad de Medicina, Universidad Católica del Maule, Talca, Chile; ^4^Servicio de Neurocirugía, Hospital Regional de Talca, Talca, Chile

**Keywords:** vertebrobasilar dolichoectasia, cerebrovascular disorders—complications, stroke, risk factors, neuroimaging

## Abstract

**Introduction:**

Vertebrobasilar dolichoectasia, a condition characterized by increased length, volume, and curvature of vertebrobasilar system, has been linked to an increased risk of cerebrovascular diseases. However, the evidence on its morphological components and risk factors for these diseases is contradictory. The lack of consensus about its characteristics, detection, cerebrovascular complications, or risk factors highlights the need for a review that synthesizes this information. Therefore, the purpose of this study was to analyze and synthesize the literature on diagnostic and detection criteria, risk factors and cerebrovascular complications associated with vertebrobasilar dolichoectasia.

**Methods:**

A scoping review was conducted following the PRISMA-ScR statement. The search was carried out in Web of Science, PubMed, and Scopus. Data on adult population with a confirmed diagnosis of vertebrobasilar tortuosity or dolichoectasia through computed tomography or magnetic resonance imaging compared with a control group were included, and excluding studies whose participants presented another neurological pathology coexisting with the cerebrovascular disease. The information was extracted, evaluated, and synthesized to provide a concrete view of the current evidence. Additionally, methodological quality was assessed with the Newcastle-Ottawa scale.

**Results:**

Of 1,373 identified studies, 18 met the eligibility criteria, including 3,058 participants (1,055 cases and 2003 controls). Vertebrobasilar dolichoectasia components, independently or associated with cardiovascular risk factors such as hypertension, atherosclerosis, diabetes, or smoking, are associated with a higher risk of cerebrovascular disease, mainly in the posterior circulation, affecting brain regions as brainstem, cerebellum, thalamus and occipital cortex.

**Conclusion:**

The findings suggest an increased risk of cerebrovascular disease when vertebrobasilar dolichoectasia or its components are present. More studies are necessary to quantify the risk of dolichoectasia components in different types of cerebrovascular disease.

## 1 Introduction

Cerebrovascular diseases are considered a global health problem, representing the third cause of death and disability in the general population, with 11% of total deaths and 5–7% of total disability adjusted to life years (1). Although the main risk factors for this condition correspond to chronic non-transmissible diseases and habits, there are also morphological factors that increase the probability of suffering from cerebrovascular diseases, such as morphological changes or anatomical variations of intracranial arteries and their branches (2, 3).

At the brain level, anatomical variations of the arterial system can influence the risk of suffering from cerebrovascular diseases, as well as a worse prognosis. Among the anatomical variations of cerebral circulation are hypoplasia or absence of branches that form the cerebral arterial circle (4), or changes in morphological parameters, such as arterial location, length, angulation, and elongation of arteries of carotid or vertebrobasilar system (5). Regarding this morphology variants, the increase in length (dolichosis), diameter (ectasia), and arterial tortuosity in brain is termed dolichoectasia (6). This condition can be asymptomatic (7), but it may also be associated with compressive or cerebrovascular symptoms (6). Dolichoectasia characteristics include turns of blood vessels, and these changes can impact hemodynamic flow, producing turbulence that may lead to thrombus formation or vascular lesions (5). The relationship between arterial tortuosity and chronic, ischemic or hemorrhagic vascular pathologies has been also documented in others arterial systems, such as retinal arteries (8), and coronary circulation (9).

**Figure 1:**
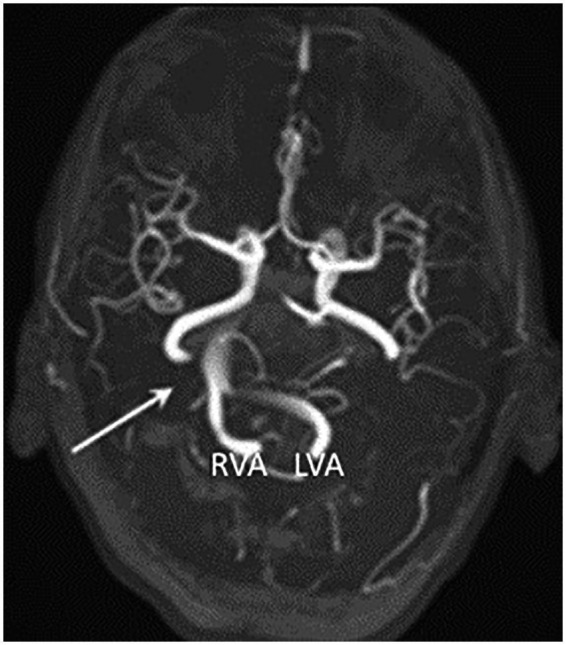
Case report of vertebrobasilar dolichoectasia published by Wang et al. (10), showing, by time-of-flight magnetic resonance angiography (TOF-MRA), a dilated and laterally displaced basilar artery (arrow) reaching the right cerebellopontine angle cistern. RVA: Right vertebral artery. LVA: Left vertebral artery. Image licensed under Creative Commons Attribution 4.0 International (CC BY 4.0), and modified for presentation in this article.

Dolichoectasia most frequently affects the brain posterior circulation, and in these cases, is known as vertebrobasilar dolichoectasia (VBD) (6). Considering the close anatomical relationship between the arteries of the posterior circulation and the brainstem with cranial nerves originating there, changes in the dimensions and location of the vertebrobasilar system can cause compressive symptoms on these structures. Cases of trigeminal neuralgia and hemifacial spasm caused by arterial compression, as well as hydrocephalus caused by obstruction of cerebrospinal fluid flow, have been documented (5, 10). VBD has also been associated with an increased risk of different types of hemorrhagic and ischemic cerebrovascular diseases of the posterior circulation, which are considered the most frequent complications associated with this condition (11). Figure 1 shows a case of VBD reported for Wang et al. (10).

The etiology of VBD is still debated, but genetic and congenital factors affecting vascular connective tissue and vascular remodeling, as well as pathologies such as hypertension and atherosclerosis, have been proposed, so it is considered that different mechanisms may coexist for the appearance of this condition (5, 6). Once established, these vascular changes can lead to alterations in arterial hemodynamic flow, forming turbulences in walls of blood vessels predisposed to damage due to genetic, congenital or acquired conditions, and leading smooth muscle atrophy, reticular deficiency, and damage to the internal elastic lamina of tortuous or dolichoectatic vessels, promoting the appearance of thrombi, emboli or hemorrhages (12).

For the detection and classification of arterial tortuosity and VBD, different imaging criteria are used, with the most common based on bifurcation height and relationship to bony landmarks considering the criteria proposed for Smoker et al. (13) (see below). However, other studies consider variables such as basilar artery length and midline deviation in centimeters (14), angulation of the vertebrobasilar junction (15), or shape of the basilar artery (16). These detection methods are typically performed manually by experienced radiologists, but recently, digital subtraction methods have been implemented, allowing for greater precision in the detection and classification of basilar arterial tortuosity (17, 18). Given the emerging variability of morphological parameters to identify and categorize the VBD, and its respective detection methods, new consensuses based on scientific evidence are needed.

Additionally, there is ongoing discussion about which risk factors, in the presence of VBD, may cause the onset of cerebrovascular diseases, where some studies suggest that the same risk factors leading to its occurrence can trigger these complications, but the data are contradictory (5). Furthermore, it is uncertain whether VBD can be considered an independent risk factor for cerebrovascular diseases, or if its different components or severity levels are associated with a greater or lesser extent with some specific type of these conditions.

There are reviews that explain possible mechanisms of origin of vertebrobasilar dolichoectasia, and its relationship with cerebrovascular diseases (5, 11, 19), but there is still no clarity about the interaction between the presence of VBD, with its components and categorizations, and different cardiovascular risk factors can influence hemodynamic flow of vertebrobasilar arterial system to lead to cerebrovascular diseases. In this sense, an analysis of these different factors through a synthesis of the evidence will improve understanding of how these factors may together contribute to the pathogenesis of ischemic or hemorrhagic cerebrovascular diseases. Considering that the most frequent complications of VBD are cerebrovascular diseases, and that different cardiovascular risk factors may contribute to the cause of VBD and influence the development of these diseases, along with the current lack of consensus on detection, neurovascular complications, and associated risk factors highlights the need for research that synthesizes the existing scientific literature. Therefore, the purpose of this review is to analyze and synthetize the literature on diagnostic and detection criteria, along with the risk factors and complications associated with VBD. A review with a broad scope and a systematic approach may contribute to the unification of criteria, and provide information that can be useful for strategies to prevent cerebrovascular diseases by controlling risk factors associated with patients with VBD, and guide future research on the detection of cerebrovascular diseases, associated risk factors, and treatment strategies.

## 2 Methods

### 2.1 Review design

A systematic review was conducted following the recommendations of the PRISMA-ScR statement (20). All analyses were based on published studies, so ethical approval and participants consent were not required.

### 2.2 Eligibility criteria

Studies were included following the PECOS approach (Population, Exposure, Comparison, Outcome, Study design): (i) Population of interest: adult population, without distinction of sex, race, or ethnicity. (ii) Exposure: presence of basilar arterial tortuosity and cerebrovascular affections. (iii) Comparison: data from the same study subjects in stages prior to the establishment of arterial tortuosity, subjects without arterial tortuosity as a control group, or subjects with arterial tortuosity without neurovascular pathologies. (iv) Outcome: cerebrovascular affection characteristics and cardiovascular risk factors. (v) Study design: Randomized controlled trials, descriptive and observational studies. Studies with the following characteristics were excluded: (i) Participants with that presented a neurological pathology coexisting with the cerebrovascular disease or pre-existing neurological pathologies. (ii) Case studies. (iii) Studies without a description of the comparison group. (iv) Absence of full text, such as conference presentations. (v) Duplicate studies.

### 2.3 Information sources and search strategy

The search was conducted in PubMed, Scopus, and Web of Science. The terms used in the article search were selected according to the research question and main objective, considering basilar tortuosity, neurovascular complications, and imaging techniques used for detection. With the aim of obtaining a broad initial data set and subsequently filtering it by applying the eligibility criteria, the search string was: (“vertebrobasilar dolichoectasia” OR “basilar dolichoectasia” OR “tortuosity”) AND (“neurovascular” OR “stroke” OR “ischemia” OR “hemorrhagic”) AND (“magnetic resonance” OR “MRI” OR “neuroimaging” OR “computed tomography” OR “CT”). Additionally, a manual search of the references of the selected articles was performed to identify possible includible studies.

### 2.4 Selection of sources of evidence

**Figure 2:**
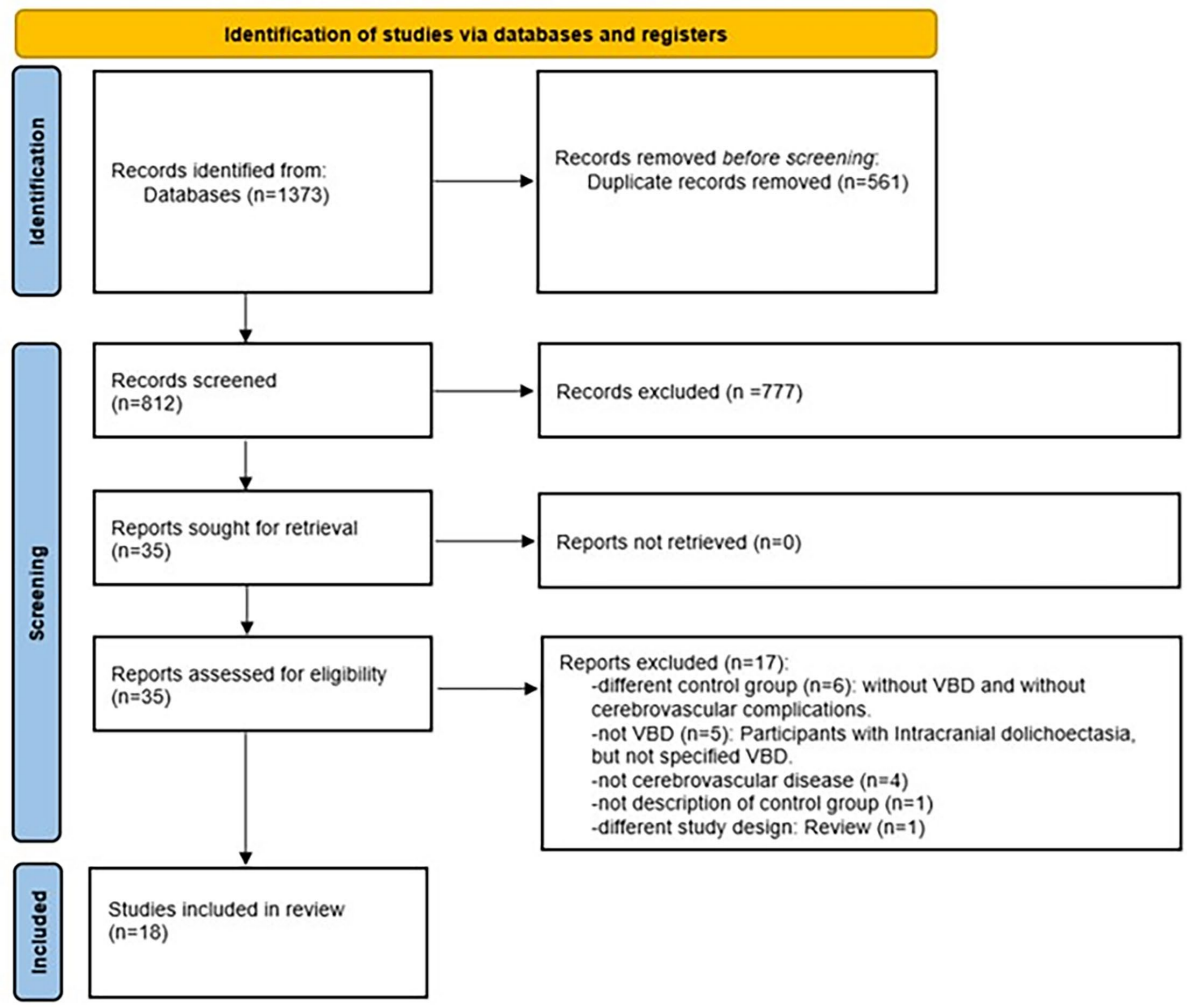
Flowchart for selection of evidence sources. VBD, vertebrobasilar dolichoectasia.

The selection process was carried out independently by two researchers (JL-F and JV-P). This process was conducted using the automated systematic review manager Rayyan^1^ (21). In the first stage (identification), the search strategy was applied to the databases, and duplicates were removed. In the second stage (screening), titles and abstracts were read, and studies that potentially met the inclusion criteria were selected for full-text reading to apply the eligibility criteria. In case of disagreements between the researchers, where consensus could not be reached, a third researcher (JM.-V) was consulted. Additionally, to expand the search and identify potentially includable articles, the reference lists of the articles included in the review were examined. Figure 2 summarizes the search strategy and selection process.

### 2.5 Data charting process

Two researchers (JM-V and FP-R) defined the most important variables to be extracted and generated a general Ad/Hoc information collection table for this study. One author completed the table with information from the included studies, and a second author checked the information, reviewing each article to corroborate the information and make clarifications when necessary. In case of discrepancies about the extracted data, a third reviewer (FO-C) was consulted.

### 2.6 Data items

The data extracted in the collection tables included demographic characteristics of the participants in the included studies, specific aspects of basilar artery tortuosity such as measured morphologic characteristics, diagnostic criteria, detection methods, and possible classifications, as well as documented cerebrovascular pathologies and associated risk factors.

### 2.7 Critical appraisal of individual sources of evidence

Included studies were subjected to methodological quality assessment by two researchers independently (JV-P and JL-F) using the Newcastle-Ottawa Scale (NOS) (22). The NOS assigns points/stars to the study considering the selection of study groups, comparability, and exposure/outcome of interest for the studies, with a maximum score of nine stars. Studies that scored 0–3 stars were considered to have low methodological quality, studies that scored 4–6 stars were considered to have medium methodological quality, and studies with a score of 7 stars or higher were considered to have high methodological quality. Disagreements were resolved by consensus or reviewed by a third researcher (JM-V). Following this evaluation, the studies that were rated as high quality were subjected to a subgroup analysis.

### 2.8 Synthesis of results

The selected and collected data in the general table were subsequently grouped into different *ad hoc* tables to generate comparisons and facilitate visualization. The main table included morphological characteristics of VBD, related cerebrovascular diseases, compromised brain structure, and summarized findings of each study (Table 1). Other important information of included studies, such as characteristics of study groups (age, sex, comorbidities, or cardiovascular risk factors), and the results of the methodological quality assessment using the Newcastle-Ottawa were incorporated on Supplementary material. One researcher incorporated the data from each Table (AF-F), and a second researcher corroborated each included data (JM-V). Finally, a qualitative analysis of the information obtained in the different studies was conducted to obtain a synthesis and generate conclusions from the literature.

**Table 1:** Sample characteristics of participants of included studies.

Author (year)	*n*	Group definition	Age (years)	Sex (M/F)	Comorbidities/cardiovascular risk factors
Cao et al. (27)	101	Cases (20): poor 90-day outcome post pontine infaction	70.5 (60–78)	11/9	Hypertension (83–82%), diabetes mellitus (46–45.5%), dyslipidemia (37–36.6%), smoking (24–23.7%) and alcohol consumption (14–13.86%)
		Controls (81): Good 90-day outcome post pontine infarction	63.0 (56–76)	48/33	
Cao et al. (14)	113	Cases (39): basilar artery elongation and acute stroke	64.1 ± 10.5	25/14	Hypertension (36–92%), diabetes mellitus (10–25.6%), dyslipidemia (16–41%), smoking (14–35.9%), alcohol consumption (9–23%),
		Controls (74): acute stroke, without elongation	63.6 ± 9.9	41/33	Hypertension (58–78.4%), diabetes mellitus (39–52.7%)*, dyslipidaemia (27–36.4%), smoking (22–29.7%), alcohol consumption (9–12.16%)
Chen et al. (33)	115	Cases (22): VBD and recurrent ischemic stroke	64.9 ± 10.0	16/6	Hypertension (20–90.9%)*, diabetes mellitus (12–54.5%)* hyperlipidemia (5–22.7%) and smoking (5–22.7%), ischemic heart disease (5–22.7%)*, intracranial atherosclerosis (11–50%), extracranial atherosclerosis (3–13.6%)
		Controls (93): VBD without recurrent ischemic stroke	62.7 ± 10.8	72/21	Hypertension (65–69.9%), diabetes mellitus (26–28%), hyperlipidemia (29–31.2%), smoking (32–34.4%), and ischemic heart disease (5–5.4%), intracranial atherosclerosis (24–25.8%)*, extracranial atherosclerosis (12–12.9%)
Chi et al. (34)	465	Cases (63): VBD	66.95 ± 11.5	46/17	Hypertension (48–76.2%)*, diabetes mellitus (28–44.4%)*, dyslipidaemia (15–23.8%)*, smoking (26–41.3%)*, alcohol consumption (14–22.2%)*, atrial fibrillation (7–11.1%), dialysis (1–1.59%)*, heart disease (6–9.5%), cancer (3–4.8%)*
		Controls (402): No VBD	67.9 ± 11	250/152	Hypertension (302–75.1%), diabetes mellitus (145–36.1%), dyslipidemia (77–19.2%) Smoking (162–40.29%), alcohol consumption (80–19.9%), atrial fibrillation* (60–14.9%), dialysis (6–1.5%), heart disease (75–18.7%), cancer (20–5%)
Çoban e al. (32)	306	Cases (186): stroke (with and without VBD)	67.9 ± 11	108/78	Hypertension (101–54.3%)*, diabetes mellitus (134–72%)* and hypercholesterolemia* (77–41.4%)
		Controls (120): no stroke (with and without VBD)	68.8 ± 12.2	57/63	Hypertension (60–50%), diabetes mellitus (43–35.9%) and hypercholesterolemia (30–25%)
Del Brutto et al. (17)	98	Cases (35): VBD	64.5 ± 11.7	20/15	Hypertension (29–85.7%), diabetes mellitus (15–42.9%), hyperlipidemia (28–80%)* smoking (10–28.6%), and coronary disease (7–20%)
		Controls (63): no VBD	63.4 ± 12.2	35/28	Hypertension (56–88.9%), diabetes mellitus (40–64.5%)*, hyperlipidemia (38–60.3%) and smoking (15–23.8%), and coronary disease (10–15.9%)
Förster et al. (29)	79	Cases (38): dolichoectasia and microhemorrhage	72.8 ± 9.5	29/9	Hypertension (33–86%), diabetes mellitus (6–15.8%), hyperlipidemia (13–34.2%), smoking (2–5.3%) and Renal failure (4–10.5%), coronary disease (3–7.9%)
		Controls (41): Dolichoectasia without microhemorrhage	71.4 ± 10.4	30/11	Hypertension (35–85.4%), diabetes mellitus (5–12.2%), hyperlipidemia (13–31.7%), smoking (2–4.9%), renal failure (4–9.7%), coronary disease (8–19.5%)
Jeong et al. (15)	416	Cases (78): DEEP pontine lacunar infarct (with and without dolichoectasia)	64.4 ± 10.5	43/35	Hypertension (53–67.9%), diabetes mellitus (24–30.8%)*, hyperlipidemia (28–35.9%)*, smoking (25–32%), alcohol consumption (30–38.5%),
		Controls (338): no deep pontine lacunar infarct (with and without dolichoectasia)	56.5 ± 9.8	189/149	Hypertension (95–28.1%), diabetes mellitus (49–14.5%), hyperlipidemia (95–28.1%), smoking (80–23.6%), alcohol consumption (158–46.7%)
Kumral et al. (23)	49	Cases (31): Dolichoectasia and stroke or transient stroke	62.7 ± 7.8	24/7	Hypertension (26–83.9%)*, diabetes mellitus (8–25.8%), hypercholesterolemia (7–22.6%), smoking (7–22.6%), atherosclerotic vascular changes (22–71%)*, low flow in the basilar artery (14–45.1%)
		Controls (18): dolichoectasia without stroke or transient stroke	61.7 ± 10	12/6	Hypertension (8–44.4%), diabetes mellitus (2–11.1%), hypercholesterolemia (3–16.7%), smoking (3–16.6%) atherosclerotic vascular changes (7–38.9%), low flow in the basilar artery (7–38.9%)
Nakamura et al. (24)	481	Group 1 (24): VBD and ischemic stroke	67.7 ± 14.3	21/3	Hypertension (15–62.5%), diabetes mellitus (7–29.2%), dyslipidemia (3–12.5%), smoking (19–79.2%)*, obesity (8–33.3%), atrial fibrillation (8–33.3%)
		Group 2 (13): VBD and brain hemorrhage	56.7 ± 12.7	13/0	Hypertension (12–92.3%), diabetes mellitus (3–23.1%), dyslipidemia (4–30.8%), smoking (5–38.46%), obesity (3–23.1%)
		Group 3 (350): ischemic stroke without VBD	69.7 ± 12.0	210/140	Hypertension (227–64.8%), diabetes mellitus (16–4.6%), dyslipidemia (73–20.9%), smoking (188–53.7%), obesity (81–23.1%), atrial fibrillation (81–23.1%)
		Group 4 (94): no VBD, with brain hemorrhage	66.8 ± 12.4	58/36	Hypertension (47–50%), diabetes mellitus (16–17%) obesity (19–20.2%), dyslipidemia (18–19.1%), smoking (20–21.3%), atrial fibrillation (11–11.7%)
Osama et al. (25)	200	Group 1 (16): VBD and brain microhemorrhage	65.22 ± 12.88	135/65	Hypertension (91–45.5%), diabetes mellitus (92–46%), dyslipidemia (110–55%), smoking (95–47.5%), ischemic cardiopathy (46–23%)
		Group 2 (3): VBD without brain microhemorrhage			
		Group 3 (96): brain microhemorrhage, without dolichoectasia			
		Group 4 (85): no brain microhemorrhage, no VBD			
Park et al. (28)	182	Cases (24): VBD	68.5 ± 11.6	16/8	Hypertension (20–83.3%)*, diabetes mellitus (20–83.3%), hyperlipidaemia (8–33.3%), smoking (14–58.3%), alcohol consumption (17–70.8%), ischemic heart disease (5–20.8%), family story of stroke (6–25%)
		Controls (158): no VBD	65 ± 11.6	80/78	Hypertension (84–53.2%), diabetes mellitus (67–42.4%), hyperlipidaemia (44–27.8%)*, smoking (57–36.1%), alcohol consumption (49–31%), ischemic heart disease (16–10.1%), family story of stroke (39–24.7%)
Passero et al. (12)	80	Cases (40): VBD and stroke	62.8 ± 8.9	31/9	Hypertension (29–72.5%), diabetes mellitus (6–15%), hyperlipidemia (20–50%), smoking (17–42.5%), alcohol consumption (8–20%), history of coronary artery disease (6–15%)
		Controls (40): VBD without stroke	61.6 ± 8.6	31/9	Not reported
Ubogu et al. (30)	90	Cases (45): VBD	73.4 ± 12.5	15/30	Hypertension (32–71.1%), diabetes mellitus (8–17.8%), hyperlipidemia (14–31.1%), smoking (21–46.7%), alcohol consumption (8–17.8%), coronary heart disease (13–28.9%), peripheral vascular disease (11–24.4%), atrial fibrillation (6–13.3%)
		Controls (45): No VBD	73.1 ± 12.2	13/32	Hypertension (29–64.4%), diabetes mellitus (4–8.9%), hyperlipidemia (12–26.7%), smoking (18–40%), alcohol consumption (9–20%), coronary heart disease (5–11.1%), peripheral vascular disease (5–11.1%), atrial fibrillation (5–11.1%)
Wang et al. (26)	56	Cases (26): VBD and posterior circulation stroke	64.76 ± 5.34	19/7	Hypertension (20–77%)*, diabetes (11–42.3%), hyperlipidemia (13–50%), smoking (12–46.2%), alcohol consumption (6–23.1%), obesity (4–15.4%), heart disease (5–19.2%), posterior circulation atherosclerosis (18–69.2%)*, family history of cardiovascular disease (7–26.9%), carotid atherosclerosis (7–26.9%)
		Controls (30): VBD without posterior circulation stroke		16/14	Hypertension (11–36,7%), diabetes (7–23.3%), hyperlipidemia (12–40%), smoking (10–33.3%), alcohol consumption (4–13.3%), obesity (5–16.7%), heart disease (4–13.3%), posterior circulation atherosclerosis (7–23.3%), family history of cardiovascular disease (5–16.7%), carotid atherosclerosis (6–20%)
Wu et al. (35)	75	Cases (34): VBD, with and without stroke	58.1 ± 9.9	30/4	Hypertension (10–29.4%), diabetes mellitus (4–11.7%), hyperlipidemia (5–14.7%), smoking (8–23.5%), atherosclerosis (14–41.17%)*
		Controls (41): Stroke, without VBD	59 ± 10.4	34/7	Hypertension (28–62.3%), diabetes mellitus (17–41.5%), hyperlipidemia (19–46.3%), smoking (24–58.5%), atherosclerosis (1–2.4%)
Zhang et al. (16)	126	Group 1 (46): acute pontine infarction with basilar artery bending	61.73 ± 8.96	29/17	Hypertension (21–45.7%), diabetes mellitus (27–58.7%)*, smoking (17–37%), hypercholesterolemia (23–50%), coronary heart disease (8–17.4%), alcohol consumption (6–13%), age ≥ 65 (24–52.2%),
		Group 2 (42): acute pontine infarction without basilar artery bending	62.36 ± 10.07	27/15	Hypertension (12–26.6%), diabetes mellitus (6–14.3%), smoking (5–11.9%), hypercholesterolemia (6–14.3%), coronary heart disease (9–21.4%), alcohol consumption (5–11.9%), Age ≥ 65 (8–19%)
		Group 3 (38): no acute pontine infarction	61.21 ± 9.16	23/15	Hypertension (13–34.2%), diabetes mellitus (3–7.9%), smoking (7–18.42%), hypercholesterolemia (9–23.7%), coronary heart disease (8–21.1%), alcohol consumption (4–10.5%). Age ≥ 65 (7–18.4%)
Zheng et al. (31)	26	Cases (15): VBD and ischemic stroke	61.0 ± 11.6	14/1	Hypertension (14–93.3%), diabetes mellitus (0–0%), hyperlipidemia (12–80%)
		Controls (11): VBD without ischemic stroke	63.2 ± 11.3	7/4	Hypertension (10–90.9%), diabetes mellitus (1–9.1%), hyperlipidemia (6–54.5%)

VBD, vertebrobasilar dolichoectasia. *Significantly higher number of participants who have this condition compared to the other study group (*p* < 0,05).

## 3 Results

### 3.1 Selection of sources of evidence

The database search strategy yielded a total of 1,373 articles. After the entire selection process, 18 studies were included in the review. Figure 2 summarizes the selection process results, with the specific number of studies excluded in each stage.

### 3.2 Characteristics of sources of evidence

#### 3.2.1 General characteristics

The total number of participants included in the selected articles was 3,058 (1878 men and 1,180 women), divided into 1,055 cases and 2003 controls. The age range of the participants was between 50.5 and 73.1 years. Table 1 summarizes demographic characteristics of the included participants and differences between groups.

#### 3.2.2 Cardiovascular risk factors

The main comorbidities and cardiovascular risk factors, considering the number of subjects and percentage of the total were: hypertension (*n* = 1773, 58%), diabetes mellitus (*n* = 949, 31.03%), dyslipidemia (*n* = 894, 29.2%), smoking (*n* = 966, 31.5%), alcohol consumption (*n* = 430, 14%), heart disease (*n* = 259, 8.4%), and obesity (*n* = 120, 3.9%).

To obtain a comparative measure of the prevalence of each of the risk factors, the range of percentages of the risk factors in cases and controls was calculated. According to this, the percentage ranges of cases compared to controls, respectively, for each condition corresponded to 29.4–93.3% vs. 26.6–90.9% for hypertension, 0–83% vs. 4.6–64.5% for diabetes mellitus, 12.5–80% vs. 14.3–60% for dyslipidemia, 5.3–79.2% vs. 4.9–58.5% for smoking, 13–70.8% vs. 11.9–46.7% for alcohol consumption, 15.4–33.3% vs. 16.7–32.1% for obesity, 13.6–69.2% vs. 2.4–25.8% for atherosclerosis, 9.5–28.9% vs. 5.4–21.4% for heart disease, and 11.1–33.3% vs. 11.1–33.3% for atrial fibrillation. Other risk factors reported in the included studies were family history of stroke and cardiovascular diseases, peripheral vascular disease and low basilar artery flow. Information regarding comorbidities was obtained from medical records for all studies, except for BMI measurements, which were performed directly. Regarding smoking and alcohol consumption habits, information was obtained from medical records or through patient interviews.

Among the diagnostic criteria for confirming the presence of risk factors, arterial hypertension was considered to be ≥140/90 mmHg in repeated measurements or use of antihypertensives (14, 15, 23–26) and one study considered a blood pressure of ≥160/90 mmHg (12). Among the studies that reported criteria for the detection of diabetes mellitus, some considered a previous diagnosis or glucose records ≥7.0 mmol/L or use of hypoglycemic agents/insulin (14, 15, 26) and others considered a medical diagnosis or fasting glucose records of ≥126 mg/dL or HbA1c ≥ 6.5%, or treatment (24, 25). For the diagnosis of dyslipidemias, the studies considered the criteria of Cholesterol ≥5.60 mmol/L, triglycerides ≥1.81 mmol/L, LDL ≥ 3.57 mmol/L or statin use (14), total cholesterol >6.2 mmol/L or LDL > 4.1 mmol/L (15), medical record of LDL ≥ 140 mg/dL or HDL < 40 mg/dL, or treatment (24), Total cholesterol ≥200 mg/dL, LDL ≥ 100 mg/dL, HDL ≤ 40 mg/dL for men, and ≤50 mg/dL for women, Triglycerides ≥150 mg/dL or treatment lipid-lowering (25), cholesterol >250 mg/dL or triglycerides >180 mg/dL (12), LDL ≥ 3.62 mmol/L and HDL < 1.03 mmol/L (26), and total cholesterol >5.17 mmol/L for hypercholesterolemia (16).

Regarding smoking and alcohol consumption, only one study reported the amount necessary to consider smoking (16), considering 1 cigarette per day continuous or accumulated for more than 6 months. The criteria for alcohol consumption for diagnosis were >400 mL of alcohol per week (12) and 45 mL of liquor ≥1 time per week (16). The most directly measured variables, such as the verification of the presence of atherosclerotic plaques, were visualized by an experienced professional.

These results highlight the heterogeneity of risk factors definitions across studies, often based only on medical history without specify the diagnostic criteria. Table 1 synthetize the characteristics of participants in each study, considering risk factors and differences between groups.

### 3.3 Synthesis of results

#### 3.3.1 Classification and detection of VBD

**Figure 3:**
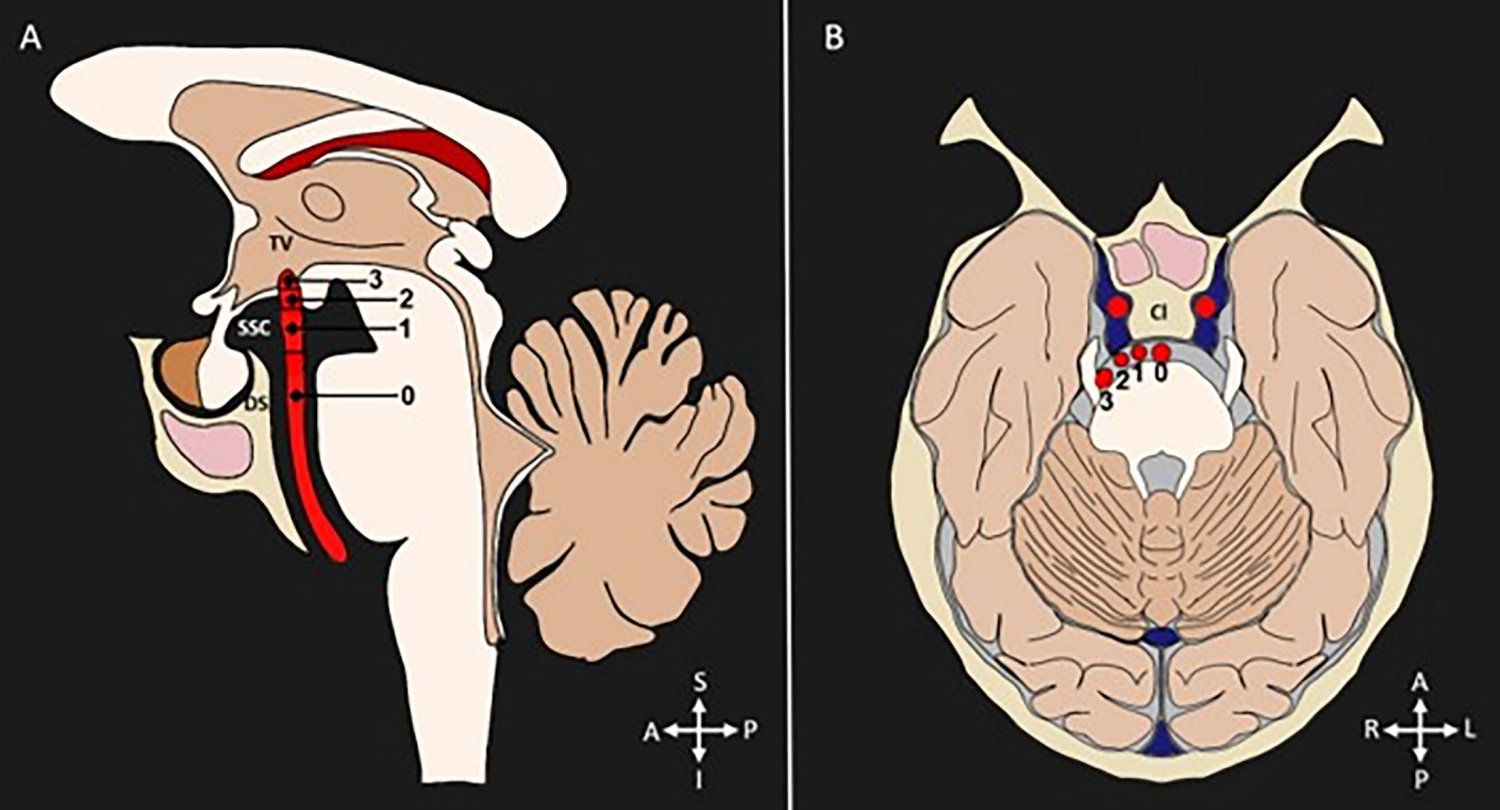
Diagnostic criteria for categorization of VBD. Diameter (≥4.5 mm). **(A)** Bifurcation height scores shown in a sagittal section: 0 = at level of dorsum sellae or below, 1 = in suprasellar cistern, 2 = at the third ventricle’s floor, 3 = indenting the floor of the third ventricle. **(B)** Lateral displacement scores shown in a transverse section: 0 = midline location, 1 = lateral to midline, and medial to the lateral margin of the clivus or sella turcica, 2 = lateral to the lateral margin of the clivus or sella turcica, 3 = cerebellopontine angle cistern location. TV, third ventricle. SSC, suprasellar cistern; DS, dorsum sellae; Cl, clivus; S, superior; I, inferior; A, anterior; P, posterior; R, right; L, left.

To detect the presence of tortuosity and VBD, most studies used the criteria established by Smoker et al. (13). This classification is based on the detection of two morphological aspects of the basilar artery: bifurcation height and laterality, each classified into four grades (grade 0 to grade 3), and a BA diameter ≥ 4.5 mm. The grades related to bifurcation are classified as follows: Grade 0: at the level of the dorsum sellae or below; Grade 1: in the suprasellar cistern; Grade 2: at the floor of the third ventricle; Grade 3: indenting the floor of the third ventricle. Regarding the laterality of the BA, the classification considers: Grade 0: in the midline; Grade 1: medial to the lateral margin of the clivus or sella turcica; Grade 2: lateral to the lateral margin of the clivus or sella turcica; Grade 3: in the cerebellopontine angle cistern. Figure 3 represents the Smoker criteria. Five articles used classifications based other variables, such as shape (16, 27) curve length (27), increases in length and diameter (17, 27), and BA angulation (15).

Regarding the detection method, the imaging techniques employed were magnetic resonance imaging techniques (MRI, HR-MRI, VWI MRI, ARMI) and computed tomography (conventional CT, angio-CT). Except for one study (17), all articles used manual methods for detecting VBD. Table 2 summarizes the findings related to morphological characteristics of the vertebrobasilar system measured in each study.

**Table 2:** Cerebrovascular complications associated with basilar arterial tortuosity.

Author (year)	-Imaging technique -criteria	Morphological variable	Pathology described	Compromised region	Main findings and associated risk factors
Cao et al. (27)	-DWI -BA ectasia: diameter >4.5 -BA dolichosis: curve length >29.5 mm or BL > 10 mm	Diameter, curvature, length and shape of BA: -BC1°: straight. BC2°: C-shaped. BC3°: inverted C-shaped. CB4°: S-shaped	Acute pontine infarction	Pontine paramedian, anterior or lateral area, multiple areas.	-BA elongation was a negative prognostic marker in acute pontine infarction (*p* = 0.009).
Cao et al. (14)	-DWI -BA curve length >29.5 mm or a bending length (BL) > 10 mm, BC1°: straight; BC2°: moderate curvature (BL ≦ 10 mm); CB3°: prominent curvature (BL > 10 mm)	Curvature, diameter and stenosis of the BA.	Recurrence of acute pontine infarction	Pontine base, deep pontine area	- > Recurrence of brainstem infarction in patients with BA elongation (*p* = 0.012). - > Risk of recurrence in age ≥ 65 years (*p* = 0.041).
Chen et al. (33)	-MRA, CTA and DSA -Smoker et al. (13)	Bifurcation height and laterality of the BA.	Ischemic stroke	Posterior circulation anterior circulation	-BA diameter ≥ 5.3 mm (*p* = 0.003) and diffuse intracranial dolichoectasia (*p* = 0.010) were independent predictors of ischemic stroke recurrence. -Previous ischemic heart disease (*p* = 0.022) was an independent predictor of ischemic stroke recurrence.
Chi et al. (34)	-MR -Smoker et al. (13)	Length, lateral displacement and diameter of BA.	Stroke	Posterior circulation	- > Prevalence of stroke in patients with VBD and VA hypoplasia.
Çoban et al. (32)	-MR -Smoker et al. (13)	Height of the basilar bifurcation, diameter, transverse position and shape of the BA.	Posterior circulation infarcts	Brainstem, cerebellum	-Type 2 or 3 elongation (*p* = 0.002) and type 2 or 3 transverse location (*p* < 0.001) of BA were more effective risk factors for posterior circulation infarcts.
Del Brutto et al. (17)	-MRI, CT. -Length and diameter increased ≥2 standard deviations from a reference population mean	Length, diameter and tortuosity index of the BA.	Intracranial atherosclerotic disease	Not registered	-No association was observed between the presence of VBD and the risk of new cerebral infarctions (*p* = 0.57). -There were no significant differences in stroke recurrence (*p* = 1.00).
Förster et al. (29)	-MR, MRA -Smoker et al. (13)	Diameter and height of the bifurcation of BA	Cerebral microbleeds and intracerebral hemorrhage	Posterior circulation, anterior circulation, thalamus, hippocampus, occipital lobe, pons, medulla oblongata, cerebellum	- > number of CMB in the posterior region than in the anterior region in patients with VBD (*p* = 0.0315). - > CMB number in men than in women (*p* = 0.0288).
Jeong et al. (15)	-MR -BA Angulation index: vertebrobasilar junction, angulation point, bifurcation point.	BA angulation	Deep Pontine Lacunar Infarction	Deep medial region of the pons	- > vertebrobasilar junction angle in patients with DPLI. (*p* = 0.047) -Age (*p* < 0.001), high blood pressure (*p* < 0.001) and Diabetes mellitus (*p* = 0.001) were associated with deep pontine lacunar infarction.
Kumral et al. (23)	-CT, MR, MRA -Smoker et al. (13)	Diameter, bifurcation height, and lateral displacement of BA	Ischemic stroke	Pons, cerebellum, midbrain, thalamus, posterior cerebral artery territory	- > frequency of diffuse atherosclerotic changes in patients with stroke (*p* = 0.028). - > Decreased blood flow in the BA (greater in patients with VBD) associated with the appearance of lesions in the distal region of the pons (*p* = 0.02). -Hypertension was associated with an increased risk of cerebrovascular events (*p* = 0.004).
Nakamura et al. (24)	-MR, MRA -Smoker et al. (13)	Bifurcation height and lateral displacement of the BA	Cerebral hemorrhage and cerebral infarction	Pons, cerebellum, anterior circulation, posterior circulation, putamen, thalamus, cerebral cortex	- > Frequency of POCI and pontine infarcts in patients with VBD (*p* = 0.005). pontine hemorrhage in patients with VBD than in patients without VBD (*p* < 0.05). -Smoking was associated with the occurrence of cerebral infarction (*p* < 0.05).
Osama et al. (25)	-CT, MR -Smoker et al. (13)	Dolichoectasia, bifurcation height and lateral displacement of the BA	Cerebral microbleeds	Posterior circulation, anterior circulation	- > Probability of suffering from cerebral microhemorrhages in patients with VBD (*p* = 0.009). - > Frequency of severe cerebral microhemorrhages in patients with VBD, (*p* = 0.007)
Park et al. (28)	-MR, MRA -Smoker et al. (13)	Dolichoectasia, bifurcation height and lateral displacement of the BA	Cerebral microbleeds	Posterior circulation, Anterior circulation	- > Incidence of cerebral microbleeds in individuals with VBD (*p* < 0.001) -Hypertension (*p* = 0.01) and leukoaraiosis (*p* < 0.001) were associated with the presence of cerebral microhemorrhages.
Passero et al. (12)	-CT, MR -Smoker et al. (13)	Bifurcation height and lateral displacement of the BA	Cerebral ischemic infarcts	Thalamus. posterior cerebral artery territory, medulla oblongata, pons, midbrain	- > incidence of infarcts in the PCA territory in patients with basilar BA diameter > 7 mm (*p* = 0.006). -Infarcts in the PCA territory were also associated with a higher height of the BA bifurcation (*p* = 0.024). -Patients with infarction presented greater vertical elongation of the BA (*p* = 0.025). - > Risk of ischemic events in patients with VBD and atherosclerosis in the circulation (*p* = 0.0006). -Atherosclerosis (*p* = 0.0006) and arterial hypertension (*p* = 0.064) were associated with the occurrence of cerebral ischemic infarcts.
Ubogu et al. (30)	-MRA, CT- -Smoker et al. (13)	Dolichoectasia	(DCP) and mortality	Posterior circulation	-VBD was associated with the development of PCD (*p* = 0.0001) and higher mortality (*p* = 0.018)
Wang et al. (26)	-MR, MRA, CT -Smoker et al. (13)	Dolichoectasia, lateral displacement and height of the bifurcation of the BA	Posterior circulation infarction	Thalamus, corpus callosum, occipital lobe, midbrain, pons. medulla oblongata	-The height of the BA bifurcation (*p* = 0.033) was associated with the occurrence of infarction in the posterior region in patients with VBD. -Hypertension (*p* = 0.005) and atherosclerosis in the posterior circulation (*p* = 0.012) were associated as risk factors for the occurrence of posterior circulation infarction in the presence of VBD.
Wu et al. (35)	-CT, MR, MRA -Smoker et al. 1986	Characteristics of the vascular wall of BA	Ischemic stroke	Posterior circulation	- > frequency of atherosclerotic plaques in patients with stroke and VBD (54.5%) than in patients with VBD without stroke (8.3%) (*p* = 0.011). - < degree of atherosclerosis in patients with VBD and stroke than in patients without VBD with stroke (*p* = 0.011).
Zhang et al. (16)	-MRA -Basilar artery curvature: LB1°: 1.02–2.68 mm; LB2°: 2.69–3.76 mm; LB3°: 3.77–7.25 mm	Curvature and length of the BA	Ischemic pontine infarction	Posterior circulation	-LB°3 was an independent risk factor for the occurrence of pontine infarction (OR = 2.74). -Advanced age, smoking (*p* = 0.041), hypertension (*p* = 0.039), elevated homocysteine levels (*p* = 0.038), history of high cholesterol (*p* = 0.016) and type 2 diabetes mellitus (*p* < 0.001) were associated with the occurrence of pontine infarcts.
Zheng et al. (31)	-MR -Smoker et al. (13)	Dolichoectasia, bifurcation height and lateral BA displacement	Ischemic stroke	Pons, cerebellum, thalamus, occipital lobe	- > BA diameter in patients with stroke than in patients without stroke (*p* < 0.001). - > Difference in BA bifurcation height in subjects with stroke (*p* = 0.002).

BA, basilar artery; VBD, vertebrobasilar dolichoectasia; VA, vertebral artery; CMB, cerebral microbleeding; DPLI, deep pons lacunar infarction; POCI, posterior circulation infarct; PCA, posterior cerebral artery; PCD, posterior circulation dysfunction; DWI, diffusion weighted image; CT, computed tomography; CTA, computerized tomography angiography; MR, magnetic resonance; MRA, magnetic resonance angiography; DSA, digital subtraction angiography; OR, odd ratio; CI, confidence interval.

#### 3.3.2 Cerebrovascular complications and anatomical structures

Among the included studies, most reported ischemic cerebrovascular disease (CVD) as a complication. Additionally, four studies reported hemorrhages (24, 25, 28, 29), while one article described posterior circulation dysfunction and mortality associated with VBD (30). Regarding the affected anatomical structures, most complications occurred in the posterior circulation, such as pontine regions (14, 15, 24, 26, 27, 29, 31), cerebellum (23, 24, 29, 31, 32), thalamus (12, 24, 29, 31), and cerebral cortex (23, 24, 26, 31). Some studies also reported damage in the anterior circulation (24, 25, 28). Table 2 summarizes the cerebrovascular complications of the included studies along with the affected brain structures.

#### 3.3.3 Cerebrovascular complications in VBD vs. no VBD participants

Among the included studies comparing subjects with and without VBD or its components, 5 associated tortuosity or its components with a higher occurrence (16, 24, 26, 27, 32) and recurrence (14) of ischemic stroke. Regarding hemorrhagic strokes, two studies indicated a higher frequency and greater risk of severity in individuals with VBD (24, 25). Another study indicated a higher frequency of posterior circulation dysfunction and mortality in subjects with VBD (30). Table 2 shows the cerebrovascular complications among participants with VBD compared to controls without VBD.

#### 3.3.4 Symptomatic VBD vs. asymptomatic VBD

Among the included articles, 6 studies compared subjects with VBD and neurovascular complications with subjects with VBD without these complications (12, 15, 23, 26, 29, 32, 33). Between these two groups, the associated factors with the occurrence of cerebrovascular diseases were grouped into morphological characteristics of vertebrobasilar system, and cardiovascular risk factors. Regarding morphological characteristics of vertebrobasilar system, the results of the included studies show that arterial length (32), curve length and lateral displacement (16, 32), vertebrobasilar junction angle (15), bifurcation height (12, 26) were related to the occurrence of CVD. Basilar artery diameter (12, 31, 33), and vertebral artery hypoplasia (34) were also related to these complications. On the other side, the main reported risk factors associated with the occurrence of ischemic cerebrovascular complications in subjects with VBD were advanced age (14, 16), history of ischemic heart disease (33), hypertension (12, 15, 16, 27), diabetes mellitus (15, 16), smoking (16, 24), atherosclerosis (12, 16, 23, 26, 35), and history of hypercholesterolemia (16). Regarding intracerebral and subarachnoid hemorrhages, the identified risk factors were hypertension (24, 25), while for cerebral microhemorrhages, men were found to be more affected than women (29). Table 2 summarizes the main findings related to the risk factors indicated in each study and Table 1 of Supplementary material contains the morphological measurements of the included studies, with corresponding comparisons between cases and controls. Regarding this, all studies, 278 except 3 (25, 28, 35), reported these measurements, with their differences and significance.

### 3.4 Results of individual sources of evidence

Individual data from each included study, such as sample characteristics, detection criteria and methods, evaluated morphological characteristics of the vertebrobasilar system, cerebrovascular complications, and risk factors, are summarized in Tables 1, 2.

### 3.5 Critical appraisal within sources of evidence

#### 3.5.1 Methodological quality assessment

According to the NOS for cases and controls studies, seven articles were rated as high methodological quality, with a score of 7 to 8 stars (15, 16, 23–25, 32, 35), while the remaining four studies (12, 26, 29, 31) were rated as medium methodological quality with a score of 5 to 6 stars. In the selection item, only two studies met the “control selection” item (25, 32).

Regarding the results of the methodological quality assessment for cohort studies, two articles were rated as high methodological quality (14, 34), and five were rated as medium risk of bias with a score of 5 to 6 stars (17, 27, 28, 30, 33). In the selection item, in the “representativeness of the exposed cohort” section, none of the articles obtained the star, while in the outcome item, in the “sufficient follow-up time” section, only one article obtained the star (30). The methodological quality assessment of each article is available in Supplementary material.

#### 3.5.2 Subgroup analysis: high methodological quality

When analyzing only the nine studies that obtained a high methodological quality, the total number of participants was 2,231, with 530 cases and 1701 controls. The total number of men and women included was 1,375 and 856, respectively. The mean age range was 56.5–69.7 years, and the risk factors present and their respective percentages were: arterial hypertension (*n* = 1,036, 46.4%), diabetes mellitus (*n* = 355, 29.4%), dyslipidemia/hyperlipidemia (481, 21.6%), smoking (*n* = 495, 22.18%), alcohol consumption (*n* = 311, 13.9%), heart disease (*n* = 152, 6.8%), and obesity (*n* = 111, 5%).

Regarding the detection methods, cerebrovascular complications and risk factors associated, it was still observed that the majority of the studies used the Smoker et al. criteria for the detection of BVD (23–25, 32, 34, 35), and said detection was by manual evaluation by an experienced professional. The most studied morphological variables among these studies corresponded to the lateral displacement and height of the BA bifurcation (23–25, 32), BA curvature and length (14, 16), and basilar artery diameter (23, 34). The main findings that remain consistent with only this subgroup of studies are a higher recurrence of pontine infarction associated with BA curvature and elongation (14, 16), and the association of VBD with a higher risk of pontine infarcts and hemorrhages (24, 25). As isolated results of the studies, it is shown that the angle of the vertebrobasilar junction is larger in patients with deep lacunar infarction (15), that grades 2 and 3 of elongation and lateral displacement of the BA increases the risk of posterior circulation infarcts (32), a higher frequency of atherosclerotic plaques in the presence of VBD and stroke (35) and a decrease in blood flow in the posterior circulation in patients with VBD (23). Finally, the risk factors that were associated with the appearance of these cerebrovascular diseases in the presence of VBD were advanced age, arterial hypertension, diabetes mellitus, smoking and dyslipidemia.

## 4 Discussion

The objective of this review was to synthesize the existing literature related to aspects of VBD, such as its characteristics, detection, reported neurovascular complications, and associated risk factors. The main results show variability in the definition of VBD, detection methods and morphologic characteristics of vertebrobasilar system analyzed to establish differences with the control group. Regarding cerebrovascular complications, the included studies report that VBD is associated with a higher risk of occurrence, recurrence, or severity of cerebrovascular diseases, mainly ischemic, but also hemorrhagic, affecting structures irrigated by the posterior circulation, such as the brainstem, thalamus, and posterior regions of the cerebral hemispheres. Additionally, different risk factors are associated with the occurrence of these complications, including morphological factors specific to VBD, associated with BA elongation, ectasia, or others; and cardiovascular risk factors: advanced age, smoking, or the presence of comorbidities such as hypertension, diabetes mellitus, or atherosclerosis.

Regarding VBD definition, the results show conceptual differences, where although most refer to an increase in arterial length and diameter, other studies also add the presence of a tortuous course (17, 29, 32, 34), distortion (26) and changes in angulation (26, 31). Considering the results of this review, it is recommended that descriptions include morphological characteristics that may influence the risk of cerebrovascular disease, such as BA elongation, lateral displacement, diameter, angulation, location of the basilar artery bifurcation, and the vertebrobasilar junction, since these morphological parameters were related to a greater or lesser extent with the appearance of cerebrovascular diseases in subjects with VBD.

According to these morphological parameters, the studies that independently analyzed them observed that VBD is related to a worse prognosis after cerebrovascular disease (27), with a higher risk of recurrence of pontine infarction (14), and the level 2 (32), or 3 (16, 32) of lateral bending of the basilar was associated with an increased risk of ischemic cerebrovascular disease of the posterior circulation. The authors suggest that VBD alter blood flow, potentially causing damage to the vascular wall, and also affecting the perforating arteries by stretching or traction, producing ischemia in the paramedian region of the pons, or promoting the appearance of thrombi in BA, which can occlude its lumen or migrate to more distal arteries of the posterior circulation (32). The results associated with these variables agrees with the general results of VBD of this review and other studies (5, 11). Curvatures of the basilar artery can also be associated with anatomical variations that generate asymmetric flow at the level of its origin or bifurcation, such as a dominance of the vertebral artery, hypoplasia of the vertebral artery or the posterior cerebral artery (34, 36).

The included studies also found that ectasia is associated with some cerebrovascular complications, where they observed a greater diameter in people with stroke (31), and a greater incidence (12) and risk of stroke recurrence (33). General results of VBD in this review also suggest that ectasia could be involved in cerebral microbleedings, caused by a risk of rupture of blood vessels due to overstretching of their walls (16, 25, 28). Ectasia also implies a decrease in the speed of blood flow, associated with the appearance of lipid deposits and the formation of microemboli (31).

Other variables studied and associated with cerebrovascular risks were the height of the BA bifurcation, the angulation of the confluence of the vertebral arteries and the basilar artery, and of these, most of studies focused on the bifurcation height, as a measure for BA elongation, which corresponds to one of Smoker’s criteria for the identification of dolichoectasia, and it was observed that it is associated with a greater risk of posterior circulation infarct (26, 32). Angulations of the vertebrobasilar junction and basilar artery were also associated with an increased risk of deep pontine lacunar infarction in patients with VBD (15), being also important variables to consider when imaging patients with this condition.

Dolichoectasia is more frequent in the posterior circulation, and although some studies indicate that in cases of VBD the anterior circulation may also be affected by cerebrovascular disease in these cases (25, 29), the vertebrobasilar system is mainly involved. There are important morphological differences between these two arterial systems: first, the components of the posterior circulation develop embryologically differently, with an earlier formation of the carotid system, and a late and complex development of the vertebrobasilar system, since the basilar artery originates through the fusion of longitudinal neural arteries, and the vertebral arteries are formed through transverse anastomoses between intersegmental arteries (37). These differences predispose to anatomical variations such as vertebral dominance or hypoplasia of the first portion of a posterior cerebral artery, which could influence hemodynamic flow and alter the position of the basilar artery (34, 36). Second, the vertebrobasilar system has less sympathetic innervation than the carotid system, this could influence a lower ability to respond to abrupt changes in flow (17). Other possible causes of the development of VBD have been described, such as changes produced by atherosclerosis or hypertension (5). However, the existence of patients with VBD without atherosclerosis or hypertension, and the difference between the structure of blood vessels between patients with dolichoectasia with and without atherosclerosis indicates the existence of other causes, such as congenital pathologies, or other conditions that alter the remodeling of blood vessels, where matrix metalloproteases (MMPs) seem to play an important role (38).

**Figure 4:**
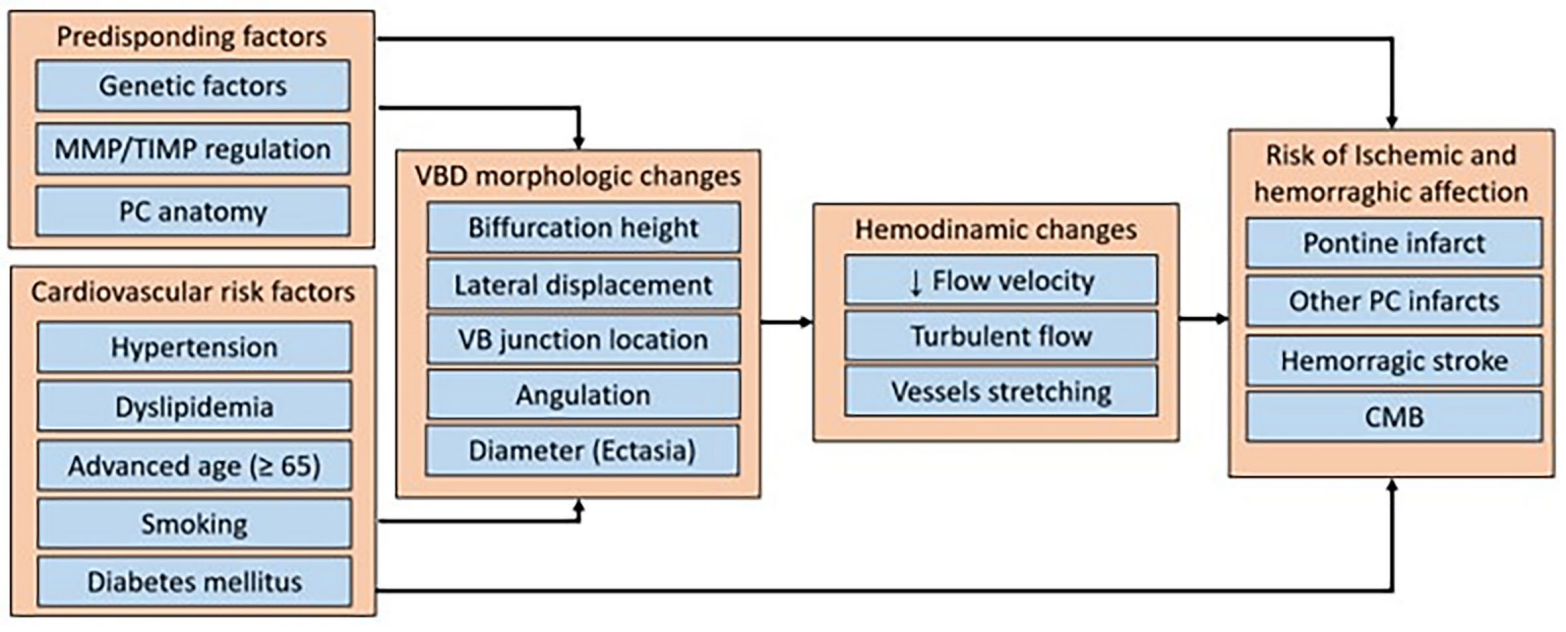
Summary diagram of the predisposing factors, cardiovascular risk factors, hemodynamic changes, and cerebrovascular diseases risk associated with vertebrobasilar dolichoectasia. MMP, matrix metalloproteinase, TIMP, tissue inhibitors of metalloproteinases; PC, posterior circulation; VBD, vertebrobasilar dolichoectasia; VB, vertebrobasilar; CMB, cerebral microbleeding.

Pico et al. (39) and Zhang et al. (38) reviewed the pathophysiology associated with morphological changes of the vertebrobasilar system in VBD, proposing that its appearance is a multifactorial phenomenon, where genetic factors affecting the extracellular matrix and vascular smooth muscle may contribute, as seen in subjects with Marfan syndrome, arterial tortuosity syndrome, Fabry syndrome or others, and conditions that affect the expression of matrix metalloproteases. These enzymes are considered potential biomarkers for VBD, since they fulfill the function of degrading extracellular proteins of the tunica media, and it has been observed that variants of the MMP-3/5A genotypes are related to the presence of intracranial dolichoectasia, and that alterations between the balance of MMP-9 and tissue inhibitor of metalloproteinases (TIMPs) are associated with VBD (38). In the included studies, the reported risk factors corresponded mainly to acquired cardiovascular pathologies, which can affect hemodynamic flow, and trigger cerebrovascular diseases in the presence of VBD, but it is also important to consider that the risk of cerebrovascular disease may be higher or lower depending on these other factors. Figure 4 summarizes the possible causal factors, the main characteristics of VBD, the changes in hemodynamic flow, and the associated cerebrovascular diseases.

The results of this review also suggest that VBD is associated with a higher risk of cerebrovascular disease and may be considered an independent risk factor for these conditions. The pathophysiological mechanisms that explain the origin of this condition are related to alterations in hemodynamic flow in the posterior circulation due to morphological changes in the tortuous vertebrobasilar system, leading to slowed blood flow in the basilar artery and tension changes in its branches (14, 27). These alterations may promote the formation of *in situ* thrombosis, which can cause pontine infarcts, as well as distal ischemia caused by embolisms, compromising structures irrigated by branches of the posterior cerebral arteries, such as the thalamus and occipital lobe (26). Changes in hemodynamic flow can also produce morphological changes in the basilar artery due to damage to the internal elastic lamina and atrophy of arterial smooth muscle, which may be related to the occurrence of hemorrhages due to injury to the affected blood vessels (5).

Vertebrobasilar tortuosity can be asymptomatic and does not always lead to the occurrence of cerebrovascular diseases (7). However, depending on morphological characteristics, such as the degree of laterality (16, 32), diameter (12, 31, 33), location of basilar artery bifurcation (12, 26), or presence of cardiovascular risk factors that influence hemodynamic flow or the condition of the blood vessel walls, the risk may be higher (15, 16, 26).

It has also been observed that among asymptomatic individuals with VBD, those with more cardiovascular risk factors, such as hypertension and atherosclerosis, presented greater tortuosity than individuals without these risk factors (7), and it is suggested that the progression of this condition and the occurrence of cerebrovascular diseases are associated with cardiovascular risk factors (12). Other risk factors identified in this review were advanced age, and smoking, which are also related to a higher risk of cerebrovascular disease in the population without BVD (1, 12). Although more studies are needed to elucidate the influence of each of these factors, both for the occurrence and progression of arterial tortuosity, the proposed relationship between arterial tortuosity and the presence of cardiovascular risk factors highlights the importance of early detection of VBD and cardiovascular risk factors control strategies to these patients.

Regarding imaging techniques and detection methods, most of the included studies used manual methods for detecting VBD. Considering recent technological advances and the importance of detecting this condition, and the effectiveness of automatic detection systems for morphological changes in other arterial systems (8, 40), the use of such tools could improve the timely detection of VBD, to implement specific cardiovascular risk prevention strategies in individuals with this condition.

Moreover, considering that emerging evidence suggests that, beyond hemodynamic alterations and degeneration of the internal elastic lamina, molecular and genetic mechanisms (including matrix metalloproteinase polymorphisms, vascular remodeling and inflammation) may contribute to the development of vertebrobasilar dolichoectasia, future research should combine high-resolution vessel-wall imaging, genetic profiling and biomarkers of vascular inflammation to clarify pathogenesis of VBD and risk of cerebrovascular disease, and consider others affections produced by VBD, such as compressive symptoms. From a translational perspective, standardized imaging criteria, prospective multicenter cohorts, and automated detection algorithms using deep learning could enable reproducible risk estimates and inform preventive strategies.

This review was conducted systematically, following an established protocol, and each stage was carried out by two researchers independently, in order to deliver results based on a structured methodological framework. Furthermore, this review contributes to knowledge in an area that should continue to be studied, and synthesizes extensive information related to detection, cerebrovascular diseases, and risk factors associated with VBD. Among the main limitations of the review is the inherent differences between the characteristics of the included studies, such as differences in the characteristics of the participants included in each study, or aspects of the methodological quality assessment that could interfere with their results. Therefore, the results of this review should be interpreted with caution.

## 5 Conclusion

The results suggest that the changes in the course, length or diameter of the vertebrobasilar system involved in VBD, independently or associated with cardiovascular risk factors such as advanced age, smoking, hypertension, and atherosclerosis, may be related to the occurrence of cerebrovascular diseases. VBD detection is performed mainly by MRI, using criteria based on diameters and relationship with bony landmarks through manual evaluation. More studies are needed to promote the timely detection of these changes according to current technological advances, to implement appropriate strategies for the prevention of cerebrovascular diseases through the control of risk factors.

## Footnotes

^1^ http://www.rayyan.ai
