# Supplementary material for: Detection, cerebrovascular complications and risk factors associated with vertebrobasilar dolichoectasia: a scoping review
Source: Front Neurol. 2025 Oct 15;16:1668912. doi: 10.3389/fneur.2025.1668912 (PMC12568028; doi:10.3389/fneur.2025.1668912)
Supplement: Supplementary file 1 [file Table_1.DOCX]

“Detection, cerebrovascular complications and risk factors associated with Vertebrobasilar Dolichoectasia: A scoping review” Supplementary Material

# Supplementary Tables

**Supplementary table 1. morphologic variables reported in included studies.**

| **-Author (year)** | **-Cases definition**  **- Controls definition** | **Morphologic variable** | **Cases** | | **Controls** | | **p value** |
| --- | --- | --- | --- | --- | --- | --- | --- |
|  |  |  | **n** | **Measure** | **n** | **Measure** |  |
| **Cao (2020)** | -Cases (20): Poor 90-day outcome post pontine infaction  -Controls (81): Good 90-day outcome post pontine infaction | BA diameter (mm) | 20 | 2.91 ± 0.44 | 81 | 2.82 ± 0.65 | 0.531 |
|  |  | Curve length (mm) | 20 | 30.61 (26.8-34.5) | 81 | 26.44 (23.57-30.10) | **0.012** |
|  |  | BA length (mm) | 20 | 28.39 (25.6-31.4) | 81 | 26.04 (22.95-28.64) | **0.039** |
|  |  | Bending length (mm) | 20 | 4.12 (3-7) | 81 | 0 (0, 4.74) | **0.011** |
| **Cao (2021)** | -Cases (39): BA elongation and acute stroke  -Controls (74): Acute stroke, without elongation | BA diameter: (mm) | 39 | 3.13 ± 0.74 | 74 | 2.76 ± 0.54 | **0.008** |
|  |  | No BA curve: n (%) | 39 | 7 (17.9) | 74 | 40 (54.1) | **<0.001** |
|  |  | Moderate curve, n (%) | 39 | 30 (76.9) | 74 | 34 (45.9) |  |
|  |  | Prominent curve, n (%) | 39 | 2 (5.1) | 74 | 0 |  |
| **Chen (2019)** | -Cases (22): VBD and recurrent ischemic stroke  -Controls (93): VBD without recurrent ischemic stroke | BA diameter ≥ 5.3 mm, n (%) | 22 | 8 (36.4) | 93 | 4 (4.3) | **<0.001** |
|  |  | BA bifurcation height = 3°, n (%) | 22 | 4 (18.2) | 93 | 3 (3.2) | **0.032** |
|  |  | BA laterality ≥ 2°, n (%) | 22 | 4 (18.2) | 93 | 15 (16.3) | 1 |
| **Chi (2019)** | -Cases (63): VBD  -Controls (402): No VBD | Vertebral artery hypoplasia, n (%) | 63 | 50 (79) | 402 | 117 (29) | **< 0.001** |
| **Çoban (2015)** | -Cases (186): Stroke (with and without VBD)  -Controls (120): No Stroke (with and without VBD) | Straight BA, n (%) | 186 | 109 (58.6%) | 120 | 100 (83.3%) | **<0.001** |
|  |  | C shaped BA, n (%) | 186 | 16 (8.6%) | 120 | 3 (2.5%) | **0.031** |
|  |  | C shaped BA, n (%) | 186 | 52 (28.0%) | 120 | 16 (13.3%) | **0.003** |
|  |  | C shaped BA, n (%) | 186 | 9 (4.8%) | 120 | 1 (0.8%) | 0.095 |
|  |  | BA Bifurcation height = 1°, n (%) | 186 | 123 (66.1%) | 120 | 111 (92.5%) | **<0.001** |
|  |  | BA Bifurcation height = 2°, n (%) | 186 | 49 (26.3%) | 120 | 9 (7.5%) | **<0.001** |
|  |  | BA Bifurcation height = 3°, n (%) | 186 | 14 (7.5%) | 120 | 0 (0%) | **0.002** |
|  |  | BA laterality = 1°, n (%) | 186 | 108 (58.4%) | 120 | 107 (89.2%) | **<0.001** |
|  |  | BA laterality = 2°, n (%) | 186 | 52 (28.1%) | 120 | 13 (10.8%) | **<0.001** |
|  |  | BA laterality = 3°, n (%) | 186 | 25 (13.5%) | 120 | 0 (0%) | **<0.001** |
|  |  | Right VA Diameter°, (mm) | 186 | 1.7 (0.5–5.8) | 120 | 2.0 (0.9–4.5) | 0.649 |
|  |  | Left VA Diameter°, (mm) | 186 | 2.3 (0.5–5.1) | 120 | 2.3 (1.0–3.5) | 0.852 |
|  |  | Right VA angle (°) | 186 | 32.0 (6.0–79.0) | 120 | 36.0 (4.0–98.0) | 0.255 |
|  |  | Left VA angle (°) | 186 | 30.5 (5.0–123.0) | 120 | 29.5 (4.0–63.0) | 0.018 |
| *Del Brutto (2021) | -Cases 1 (6): symptomatic V4  -Cases 2 (18): symptomatic BA  -Controls 1 (158): Nonsymptomatic V4  -Controls 2 (80): Nonsymptomatic BA | V4 proximal diameter (mm) | 6 | 3.9 ± 0.5 | 158 | 3.4 ± 0.8 | 0.20 |
|  |  | V4 distal diameter (mm) | 6 | 3.1 ± 0.5 | 158 | 2.5 ± 0.7 | **0.08** |
|  |  | V4 Length (mm) | 6 | 44.0 ± 4.2 | 158 | 39.2 ± 5.4 | 0.05 |
|  |  | VA Tortuosity Index | 6 | 1.1 (0.1) | 158 | 1.1 (0.1) | 0.42 |
|  |  | V4 dolichoectasia prevalence, (%) | 6 | 0 (0.0) | 158 | 16/158 (10.1) | 1.00 |
|  |  | BA proximal diameter (mm) | 18 | 3.3 ± 0.9 | 80 | 3.3 ± 0.6 | 0.92 |
|  |  | BA distal diameter (mm) | 18 | 2.8 ± 0.7 | 80 | 2.8 ± 0.6 | 0.93 |
|  |  | BA Length (mm) | 18 | 35.4 ± 7.0 | 80 | 30.8 ± 5.4 | **<0.01** |
|  |  | BA tortuosity index, med (IQR) | 18 | 1.2 (0.0) | 80 | 1.1 (0.1) | **0.01** |
|  |  | BA dolichoectasia prevalence, n (%) | 18 | 5 (27.8) | 80 | 7 (8.8) | **0.04** |
| Förster (2020) | -Cases (38): VBD and microhemorrhage  -Controls (41): VBD without microhemorrhage | BA diameter (mm) | 38 | 7.4 ± 5.5 | 41 | 6.0 ± 3.8 | 0.19 |
|  |  | BA bifurcation height score, med (IQR) | 38 | 2 (1–3) | 41 | 2 (1–2) | 0.25 |
|  |  | BA laterality score, med (IQR) | 38 | 5 (4–8) | 41 | 5 (4–8) | 0.72 |
| Jeong (2015) | -Cases (78): Deep pontine lacunar infarct (with and without dolichoectasia)  -Controls (338): No Deep pontine lacunar infarct (with and without dolichoectasia | BA anterior angulated, n (%) | 78 | 76 (97.4) | 338 | 333 (85.5) | 0.5 |
|  |  | BA right lateral angulated, n (%) | 78 | 44 (56.4) | 338 | 223 (66.1) | 0.11 |
|  |  | VB junction angle index (°) | 78 | 19.3 ± 7.1 | 338 | 16.5 ± 4.5 | **0.047** |
|  |  | Angulation point angle index (°) | 78 | 95.9 ±8.2 | 338 | 98.2 ± 6.8 | **0.009** |
|  |  | Angle index division point (°) | 78 | 14.6 ± 4.8 | 338 | 14.2 ± 4.2 | 0.646 |
|  |  | Triangle area index (mm^2^) | 78 | 48.8 ± 20.6 | 338 | 49.1 ± 22.1 | 0.898 |
|  |  | Triangle height index (mm) | 78 | 3.7 ± 1.2 | 338 | 3.6 ± 1.1 | 0.429 |
|  |  | VB junction to BA bifurcation distance (mm) | 78 | 27.4 ± 4.4 | 338 | 27.1 ± 5 | 0.727 |
|  |  | VA right dominance (%) | 78 | 16 (20.5) | 338 | 17 (5) | **<0.001** |
|  |  | VA left dominance (%) | 78 | 33 (42.3) | 338 | 16 (4.7) |  |
|  |  | No VA dominance (%) | 78 | 29 (37.2) | 338 | 305 (90.3) |  |
| Kumral (2005) | -Cases (31): Dolichoectasia and stroke or transient stroke  -Controls (18): Dolichoectasia without stroke or transient stroke | BA diameter ≥ 7 mm, n (%) | 31 | 17 (55) | 18 | 7 (39) | 0.19 |
|  |  | Bifurcation height ≥ 35 mm, n (%) | 31 | 27 (87) | 18 | 14 (78) | 0.39 |
|  |  | BA Laterality, n (%) | 31 | 22 (68) | 18 | 9 (50) | 0.21 |
|  |  | BA Low flow, n (%) | 31 | 14 (45) | 18 | 4 (22) | 0.1 |
| *Nakamura (2010) | -Cases, group 1 (24): VBD and ischemic stroke  -Controls, group 2 (13): VBD and brain hemorrhage | BA diameter (mm) | 24 | 5 ± 0.4 | 31 | 5.1 ± 0.5 | > 0.01 |
|  |  | BA bifurcation height (score) | 24 | 1.6 ± 0.6 | 31 | 2.1 ± 0.3 | **< 0.01** |
|  |  | BA laterality (score) | 24 | 1.4 ± 0.7 | 31 | 2.2 ± 0.9 | **< 0.01** |
|  |  | VA diameter (mm) | 24 | 4.5 ± 0.3 | 31 | 4.6 ± 0.4 | > 0.01 |
| Passero (1998) | -Cases (40): VBD and stroke  -Controls (40): VBD without stroke | BA diameter ≥ 7, n (%) | 40 | 13 (32.5) | 40 | (22.5) | 0.31 |
|  |  | BA bifurcation ≥ 2 (score) , n (%) | 40 | 34 (85) | 40 | (60) | **0.025** |
|  |  | BA laterality ≥ 2 (score), n (%) | 40 | 32 (80) | 40 | (72.5) | 0.84 |
| Ubogu (2004) | -Cases (45): VBD  -Controls (45): No VBD | BA dolichoectasia, n (%) | 45 | 18 (40) | 45 | -- | NR |
|  |  | Bilateral VA dolichoectasia, n (%) | 45 | 10 (22) | 45 | -- | NR |
|  |  | Unilateral VA dolichoectasia, n (%) | 45 | 7 (16) | 45 | -- | NR |
|  |  | BA and single VA dolichoectasia, n (%) | 45 | 2 (4) | 45 | -- | NR |
| Wang (2018) | -Cases (26): VBD and posterior circulation stroke  -Controls (30): VBD without posterior circulation stroke | BA diameter ≤ 6mm, n (%) | 26 | 13 (50) | 30 | 21 (70) | 0.126 |
|  |  | BA diameter > 6mm, n (%) | 26 | 13 (50) | 30 | 9 (30) |  |
|  |  | BA bifurcation height = 1°, n (%) | 26 | 2 (7.7) | 30 | 8 (26.7 | **0.033** |
|  |  | BA bifurcation height = 2°, n (%) | 26 | 11 (42.3) | 30 | 16 (53.3) |  |
|  |  | BA bifurcation height = 3°, n (%) | 26 | 13 (50) | 30 | 6 (20) |  |
|  |  | BA laterality = 1°, n (%) | 26 | 6 (23.1) | 30 | 8 (26.7) | 0.366 |
|  |  | BA laterality = 2°, n (%) | 26 | 15 (57.7) | 30 | 12 (40) |  |
|  |  | BA laterality = 3°, n (%) | 26 | 5 (19.2) | 30 | 10 (33.3) |  |
|  |  | BA length, ≤ 35 mm, n (%) | 26 | 11 (42.3) | 30 | 12 (40) | 0.861 |
|  |  | BA length, < 35 mm, n (%) | 26 | 15 (57.7) | 30 | 18 (60) |  |
|  |  | Intracranial VA length, ≤ 25 mm, n (%) | 26 | 13 (50) | 30 | 11 (36.7) | 0.315 |
|  |  | Intracranial VA length, ≤ 25 mm, n (%) | 26 | 13 (50) | 30 | 19 (63.3) |  |
| Zhang (2014) | Cases group 1 (46): acute pontine infarction with basilar artery bending  -Control group Group 1 (42): acute pontine infarction without basilar artery bending  -Control Group 2 (38): no acute pontine infarction | BA lateral displacement (mm) | 46 | 5.54 ± 3.06 | 42 | 3.20 ± 1.44 | **0.031** |
|  |  |  |  |  | 38 | -- |  |
|  |  | VA dominance, n (%) | 46 | 33 (71.7) | 42 | 23 (54.8) | **0.039** |
|  |  |  |  |  | 38 | 21 (55.2) |  |
|  |  | BA diameter (mm) | 46 | 3.98 ± 0.52 | 42 | 3.73 ± 0.61 | 0.721 |
|  |  |  |  |  | 38 | 3.82 ± 0.47 |  |
| Zheng (2023) | -Cases (15): VBD and ischemic stroke  -Controls (11): VBD without ischemic stroke | BA diameter (mm) | 15 | 6.57 ± 1.00 | 11 | 5.06 ± 0.50 | **<.0.001** |
|  |  | BA bifurcation height = 1°, n (%) | 15 | 0 (0) | 11 | 1 (9.1) | **0.002** |
|  |  | BA bifurcation height = 2°, n (%) | 15 | 6 (40%) | 11 | 10 (90.9) |  |
|  |  | BA bifurcation height = 3°, n (%) | 15 | 9 (60) | 11 | 0 (0) |  |
|  |  | BA laterality = 1°, n (%) | 15 | 6 (40) | 11 | 5 (45.5) | 0.763 |
|  |  | BA laterality = 2°, n (%) | 15 | 6 (40) | 11 | 5 (45.5) |  |
|  |  | BA laterality = 2°, n (%) | 15 | 3 (20%) | 11 | 1 (9.1) |  |

**Supplementary table 2. Methodological quality assessment, cases and controls studies**

| **Author (year)** | **Selection** | | | | **Comparability** | **Exposure** | | | **Score** | **Classification** |
| --- | --- | --- | --- | --- | --- | --- | --- | --- | --- | --- |
|  | **Case Definition** | **Representativity** | **Selection of controls** | **Control definition** | **Control of confoundments** | **Ascertainment of exposure** | **ascertainment for cases and controls** | **Non-Response rate** |  |  |
| **Çoban (2015)** | * | * | * | * | * | * | * |  | 8 | High quality |
| **Förster (2018)** | * | * |  |  | * | * | * |  | 5 | Medium quality |
| **Jeong (2015)** | * | * |  | * | * | * | * | * | 7 | High quality |
| **Kumral (2005)** | * | * |  | * | * | * | * | * | 7 | High quality |
| **Nakamura (2018)** | * | * |  |  | ** | * | * | * | 7 | High quality |
| **Osama (2022)** | * | * | * | * | * | * | * |  | 7 | High quality |
| **Passero (1998)** | * | * |  |  | * |  | * | * | 5 | Medium quality |
| **Wang (2018)** | * | * |  |  | * | * | * | * | 6 | Medium quality |
| **Wu (2023)** | * | * |  | * | ** | * | * | * | 8 | High quality |
| **Zhang (2014)** | * | * |  | * | ** | * | * | * | 8 | High quality |
| **Zheng (2023)** | * | * |  |  | * | * | * | * | 6 | Medium quality |

**Supplementary Table 3. Methodological quality assessment, cohort studies.**

| **Autor (year)** | **Selection** | | | | **Comparability** | **Outcome** | | | **Score** | **Classification** |
| --- | --- | --- | --- | --- | --- | --- | --- | --- | --- | --- |
|  | **Representativeness of the exposed cohort** | **Selection of the non-exposed cohort** | **Ascertainment of exposure** | **Demonstration that outcome of interest was not present at start of study** | **Comparability of cohorts on the basis of the design or analysis** | **Assessment of outcome** | **Was follow-up long enough for outcomes to occur e** | **Adequacy of follow up of cohorts** |  |  |
| **Cao (2020)** |  | ***** | ***** | ***** | ***** | ***** |  | ***** | 6 | Medium quality |
| **Cao (2021)** |  | ***** | ***** | ***** | ****** | ***** |  | ***** | 7 | High quality |
| **Chi (2019)** |  | ***** | ***** | ***** | ****** | ***** |  | ***** | 7 | High quality |
| **Chen (2019)** |  | ***** | ***** |  | ***** | ***** |  | ***** | 5 | Medium quality |
| **Del Brutto (2021)** |  |  | ***** | ***** | ***** | ***** |  | ***** | 5 | Medium quality |
| **Park (2013)** |  | ***** | ***** |  | ****** | ***** |  | ***** | 6 | Medium quality |
| **Ubogu (2004)** |  | ***** | ***** |  | ***** | ***** | ***** | ***** | 6 | Medium quality |

**Supplementary table 4. Checklist of PRISMA-ScR items (Tricco et al., 2018)**

| **Section** | **Item PRISMA-ScR Checklist Item** | **Page** |
| --- | --- | --- |
| **Title** | | |
|  | 1- Identify the report as a scoping review. | 1 |
| **Abstract** | | |
| **Structured summary** | 2- Provide a structured summary that includes (as applicable) background, objectives, eligibility criteria, sources of evidence, charting methods, results, and conclusions that relate to the review questions and objectives. | 1 |
| **Introduction** | | |
| Rationale | 3-Describe the rationale for the review in the context of what is already known. Explain why the review questions/objectives lend themselves to a scoping review approach. | 2-3 |
| Objectives | 4 Provide an explicit statement of the questions and objectives being addressed with reference to their key elements (e.g., population or participants, concepts, and context) or other relevant key elements used to conceptualize the review questions and/or objectives. | 4 |
| **Methods** | | |
| Protocol and registration | 5-Indicate whether a review protocol exists; state if and where it can be accessed (e.g., a Web address); and if available, provide registration information, including the registration number. | 4 |
| Eligibility criteria | 6-Specify characteristics of the sources of evidence used as eligibility criteria (e.g., years considered, language, and publication status), and provide a rationale. | 4 |
| Information sources | 7-Describe all information sources in the search (e.g., databases with dates of coverage and contact with authors to identify additional sources), as well as the date the most recent search was executed. | 4 |
| Search | 8-Present the full electronic search strategy for at least 1 database, including any limits used, such that it could be repeated. | 4 |
| Selection of sources of evidence | 9-State the process for selecting sources of evidence (i.e., screening and eligibility) included in the scoping review. | 5 |
| Data charting process | 10-Describe the methods of charting data from the included sources of evidence (e.g., calibrated forms or forms that have been tested by the team before their use, and whether data charting was done independently or in duplicate) and any processes for obtaining and confirming data from investigators. | 5 |
| Data items | 11-List and define all variables for which data were sought and any assumptions and simplifications made. | 5 |
| Critical appraisal of individual sources of  evidence | 12-If done, provide a rationale for conducting a critical appraisal of included sources of evidence; describe the methods used and how this information was used in any data synthesis (if appropriate). | - |
| Summary measures | 13-Not applicable for scoping reviews | - |
| Synthesis of results | 14-Describe the methods of handling and summarizing the data that were charted. | - |
| Risk of bias across studies | 15-Not applicable for scoping reviews. | - |
| Additional analyses | 16-Not applicable for scoping reviews. | 5 |
| **Results** | | |
| Selection of sources of evidence | 17-Give numbers of sources of evidence screened, assessed for eligibility, and included in the review, with reasons for exclusions at each stage, ideally using a flow diagram. | 6 |
| Characteristics of sources of evidence | 18-For each source of evidence, present characteristics for which data were charted and provide the citations. | 6 |
| Critical appraisal within sources of evidence | 19-If done, present data on critical appraisal of included sources of evidence (see item 12). | - |
| Results of individual sources of evidence | 20-For each included source of evidence, present the relevant data that were charted that relate to the review questions and objectives. | 6-8 |
| Synthesis of results | 21-Summarize and/or present the charting results as they relate to the review questions and objectives. | 8 |
| Risk of bias across studies | 22-Not applicable for scoping reviews. | - |
| Additional analyses | 23-Not applicable for scoping reviews. | Sup. Material |
| **Discussion** | | |
| Summary of evidence | 24-Summarize the main results (including an overview of concepts, themes, and types of evidence  available), link to the review questions and objectives, and consider the relevance to key groups. | 9-11 |
| Limitations | 25-Discuss the limitations of the scoping review process. | 12 |
| Conclusions | 26 Provide a general interpretation of the results with respect to the review questions and objectives, as  well as potential implications and/or next steps. | 12 |
| Funding | 27 Describe sources of funding for the included sources of evidence, as well as sources of funding for the scoping review. Describe the role of the funders of the scoping review. | 12 |
